# Advanced development of biomarkers for immunotherapy in hepatocellular carcinoma

**DOI:** 10.3389/fonc.2022.1091088

**Published:** 2023-01-16

**Authors:** Xuenan Peng, Caifeng Gong, Wen Zhang, Aiping Zhou

**Affiliations:** Department of Medical Oncology, National Cancer Center/National Clinical Research Center for Cancer/Cancer Hospital, Chinese Academy of Medical Sciences and Peking Union Medical College, Beijing, China

**Keywords:** hepatocellular carcinoma, immunotherapy, combination therapy, biomarker, tumor immune microenvironment

## Abstract

Hepatocellular carcinoma (HCC) is the most common liver cancer and one of the leading causes of cancer-related deaths in the world. Mono-immunotherapy and combination therapy with immune checkpoint inhibitors (ICIs) and multitargeted tyrosine kinase inhibitors (TKIs) or anti-vascular endothelial growth factor (anti-VEGF) inhibitors have become new standard therapies in advanced HCC (aHCC). However, the clinical benefit of these treatments is still limited. Thus, proper biomarkers which can predict treatment response to immunotherapy to maximize clinical benefit while sparing unnecessary toxicity are urgently needed. Contrary to other malignancies, up until now, no acknowledged biomarkers are available to predict resistance or response to immunotherapy for HCC patients. Furthermore, biomarkers, which are established in other cancer types, such as programmed death ligand 1 (PD-L1) expression and tumor mutational burden (TMB), have no stable predictive effect in HCC. Thus, plenty of research focusing on biomarkers for HCC is under exploration. In this review, we summarize the predictive and prognostic biomarkers as well as the potential predictive mechanism in order to guide future research direction for biomarker exploration and clinical treatment options in HCC.

## Introduction

**Figure 1:**
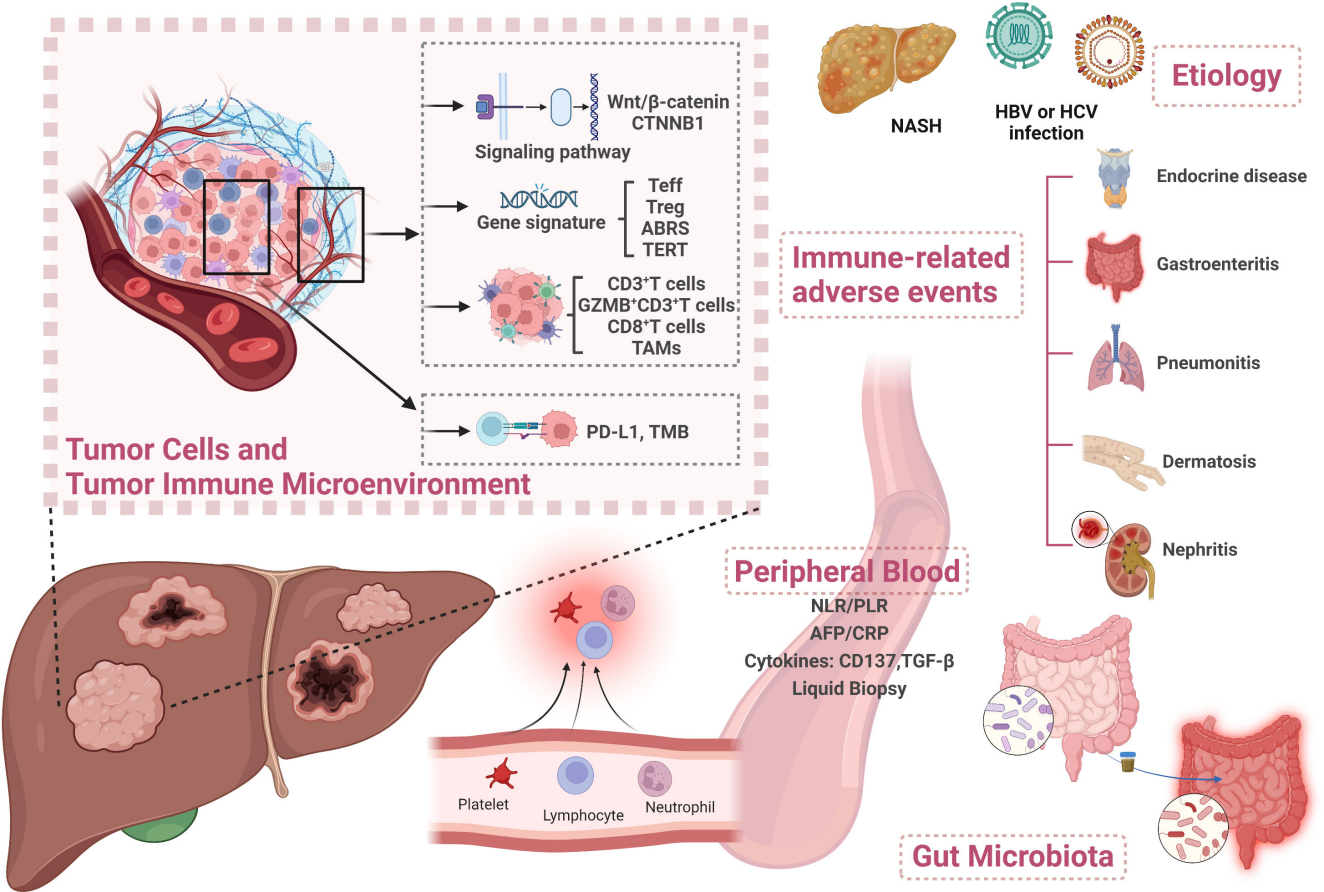
Overview of biomarkers for predicting treatment response to immunotherapy in HCC. Created with BioRender.com. Teff, T effector; ABRS, atezolizumab + bevacizumab response signature; TAM, tumor-associated macrophage; PD-L1, programmed death ligand 1; TMB, tumor mutational burden; NLR, neutrophil-to-lymphocyte ratio; PLR, platelet-to-lymphocyte ratio; AFP, alpha-fetoprotein; CRP, C-reactive protein; TGF-β, transforming growth factor-β; HBV, hepatitis B virus; HCV, hepatitis C virus; NASH, non-alcoholic steatohepatitis.

Hepatocellular carcinoma (HCC) is the most common primary liver cancer and mostly develops on a background of chronic liver disease (1). Most patients were diagnosed at an advanced stage and/or had underlying chronic liver disease, with no opportunity to receive liver resection and transplantation. Moreover, even diagnosed at an early stage, the recurrence rates remain at about 70% in 5 years after surgery (2). Systemic treatment options for advanced HCC (aHCC) by multitargeted tyrosine kinase inhibitors (TKIs) of sorafenib, lenvatinib, regorafenib, cabozantinib, and ramucirumab have improved aHCC patients’ survival in a certain degree. However, the overall survival (OS) is merely 10.7-13.6 months (3–7), far from clinical expectation. In recent years, immune checkpoint inhibitors (ICIs) including nivolumab and pembrolizumab have shown survival benefits and have been approved by the United States Food and Drug Administration (FDA) for aHCC treatment (8, 9). Since 2020, anti-programmed death 1 (anti-PD-1) antibodies such as camrelizumab and tislelizumab have been successively approved by National Medical Products Administration (NMPA) as second-line treatment regimens for HCC patients (10, 11). The IMbrave150 trial achieved an improvement in OS of up to 19.2 months with atezolizumab and bevacizumab combination therapy, making it the standard first-line treatment for aHCC (12, 13). Regrettably, the objective response rate (ORR) of combination therapy was only about 30% (14). In addition, approximately 5%–30% of patients develop ≥ grade 3 immune-related adverse events (irAEs) (14). Therefore, proper biomarkers used to predict patient clinical response and spare unnecessary toxicity are urgently needed. Although no widely accepted biomarkers have been identified currently, multidimensional analyses of potential biomarkers for immunotherapy of HCC have been under exploration. In this review, we aim to summarize the predictive and prognostic biomarkers from multiple dimensions to guide future biomarker exploration in HCC (**Figure 1**).

## Circulating biomarkers in peripheral blood

### NLR and PLR

Human neutrophils and platelets produce a host of cytokines and growth factors relevant to tumor growth and progression (15–25). Neutrophil-to-lymphocyte ratio (NLR) and platelet-to-lymphocyte ratio (PLR) have been reported as predictive factors in several cancer types (26–34). Elevated NLR and PLR were also found to be associated with poor response to transarterial chemoembolization (TACE) and sorafenib treatment in HCC (35–41). As for immunotherapy in HCC, the same predictive effect has also been reported. In a subcohort of 242 patients in the CheckMate 040 trial, patients with NLR in the low tertile showed better OS than those with medium or high tertile (*p =* 0.015) (42). A similar result was observed in PLR (*p =* 1.38e−07). Patients with complete response or partial response (CR/PR) had lower PLR than those with progressive disease (PD) (*p* = 0.05). In another cohort of 194 aHCC patients treated with nivolumab, those with baseline NLR ≥3 presented poorer progression-free survival (PFS) [11.0 vs. 7.1 weeks; HR = 1.52 (95% CI 1.11–2.07), *p* = 0.01] and OS [61.3 vs. 21.0 weeks; HR = 2.72 (95% CI 1.86–3.99), *p* < 0.001]. Moreover, a dynamic increase of NLR at 4 weeks was associated with an increased risk of death [HR = 1.79, 95% CI (1.19–2.68)]. Interestingly, in this study, NLR increased at 4 weeks also had a role in predicting hyperprogressive disease (HPD), which may guide treatment plan in an early phase (43). In a cohort of 362 HCC patients treated with mono or combination immunotherapy, patients with higher NLR and PLR at baseline were reported to have a higher incidence of portal vein thrombosis (PVT), higher Eastern Cooperative Oncology Group (ECOG) performance status, and more advanced Barcelona Clinic Liver Cancer (BCLC) stage. Significantly shorter OS and PFS were observed in patients with NLR ≥5 (OS: 7.7 vs. 17.6 months, *p* < 0.0001; PFS: 2.1 vs. 3.8 months, *p* = 0.03) and PLR ≥300 (OS: 6.7 vs.16.5 months, *p* < 0.0001; PFS: 1.8 vs. 3.7 months, *p* = 0.0006) (44). On the basis of the independent predictive role for OS of NLR and PLR, Schobert et al. found that the combination of high NLR and PLR was associated with an eightfold increased risk of death (40).

In conclusion, several analyses in different trials have demonstrated the strong survival predictive power of NLR and PLR in HCC immunotherapy and their predictive trend in treatment response. As for potential mechanisms, some reported that IL-8 and other tumor growth factors secreted by tumors may promote neutrophil recruitment (45). The increasing circulating and intratumoral neutrophils can further secrete vascular endothelial growth factor (VEGF), thereby causing higher levels of VEGF in the tumors (46) and promoting angiogenesis.

### AFP and CRP

Alpha-fetoprotein (AFP) is widely used for the surveillance, diagnosis, and prognostication of HCC. In recent decades, several studies have been conducted to explore its additional roles, such as being employed for defining HCC molecular classes or as biomarkers for HCC treatment (47–51). In a cohort of 99 patients who received nivolumab or pembrolizumab, those with AFP <400 μg/L at the beginning of ICI treatment were more likely to achieve a higher rate of CR or PR than those with AFP ≥400 μg/L (24% vs. 13%). Patients with baseline serum AFP <400 μg/L presented longer PFS (5.4 vs. 2.6 months, *p* < 0.05) and OS (21.8 vs. 8.7 months, *p* < 0.0001) (52). Moreover, a simple and easily applicable score called C-reactive protein (CRP) and AFP in Immunotherapy (CRAFITY) constructed by the analysis of 190 aHCC patients who received mono or combination immunotherapy based on CRP and AFP was recently reported (53). In this score, AFP ≥100 ng/ml and CRP ≥1 mg/dl were both assigned 1 point. Patients could achieve either 0, 1, or 2 points depending on the level of these two variables. Results showed that baseline serum AFP ≥100 ng/ml and CRP ≥1 mg/dl were independently associated with worse OS in ICI-treated patients with HCC. The median OS of patients with 0 points (CRAFITY - low) (*n* = 53), 1 point (CRAFITY - intermediate) (*n* = 75), and 2 points (CRAFITY - high) (*n* = 62) were 27.6 vs. 11.3 vs. 6.4 months (*p* < 0.001). In addition, a high CRAFITY score also predicted a worse radiological response, and the disease control rate (DCR) was 80% vs. 64% vs. 39% for a score of 0, 1, and 2, respectively (*p* < 0.001). Yang et al. further verified the CRAFITY score in TKI plus immunotherapy and lenvatinib monotherapy cohorts. A high score successfully predicts worse OS and a trend toward worse ORR and DCR (54). This simple prognostic score facilitates early survival evaluation of immunotherapy treatment and is promising to be adopted in clinical application. However, CRP is an acute-phase protein which may increase after injury or infection. Diseases which may increase CRP levels should be considered before score application.

### Cytokines

#### Transforming growth factor-β

Transforming growth factor-β (TGF-β) is known as an immunosuppressive and fibrotic cytokine. Approximately 38% of HCC patients have somatic mutations in the TGF-β pathway (55). High TGF-β levels present more aggressive tumor characteristics and may also cause T-cell exhaustion by upregulating PD-1 signaling in HCC, which demonstrates a specific immunosuppressive role of TGF-β in mediating immunotherapy resistance (56–59). Feun et al. conducted a phase 2 study of pembrolizumab in 29 patients (24 provided plasma samples) with aHCC. In the biomarker analyses, plasma TGF-β levels in responders [those with CR/PR/stable disease (SD)] were lower than those in non-responders (141.9 vs. 1,071.8 pg/ml, *p* = 0.004). Survival analysis showed that patients with plasma TGF-β ≥200 pg/ml had significantly shorter PFS (2 vs. over 25 months, *p* = 0.008) and OS (7 vs. over 25 months, *p* = 0.005), indicating that higher TGF-β levels were associated with poor treatment outcomes (60). This suggests that high plasma TGF-β may be a potential biomarker for poor treatment response and outcome to immunotherapy, which may be related to the tumor microenvironment of decreased T-cell infiltration in tumors shaped by TGF-β (61). However, the role of TGF-β in HCC is still in the exploratory stage, and its predictive value needs to be further confirmed in large-scale studies.

#### CD137

CD137, also known as 4-1BB or TNF receptor superfamily member 9 (TNFRSF9), is a member of the tumor necrosis factor family and an important costimulatory molecule in the process of T-cell activation, which can enhance the antitumor effects of T cells (62). CD137 is mainly expressed by activated CD4^+^ and CD8^+^ T cells (63), it is also found on the surfaces of NK cells, neutrophils, dendritic cells, and monocytes (64, 65). The expression of CD137 in HCC was higher than that in other types of cancer (e.g., small cell lung cancer and colorectal cancer) and was found to be expressed predominantly on exhausted PD-1^high^CD8^+^ T cells (66), as well as activated T cells in peripheral blood samples (67, 68). Preclinical studies have found a synergistic antitumor activity between PD-1/programmed death ligand 1 (PD-L1) inhibitors and activation of the CD137 signaling pathway (69). The increased number of CD137^+^CD8^+^ T cells in peripheral blood was correlated with longer disease-free survival (DFS) in patients with melanoma who were treated with ipilimumab plus nivolumab (70). A study recently conducted on 50 aHCC patients who received sintilimab (a PD-1 inhibitor) plus IBI305 reported the potential predictive role of serum CD137. Among 33 patients with serum CD137 detected, the CD137 concentration was significantly higher in patients with clinical benefit (CB, patients with CR/PR, or SD ≥12 weeks) than in those with non-CB (patients with PD or SD <12 weeks) (32.8 vs. 19.8 pg/ml, *p* = 0.034). Markedly longer PFS (14.2 vs. 4.1 months, *p* < 0.001) and OS (undefined vs. 15.6 months, *p* = 0.023) were observed in patients with high CD137 concentrations (71). However, relevant studies are mainly small sample research. Its predictive role remains to be further explored.

### Liquid biopsy

HCC exhibits significant heterogeneity from genetic aberrations and transcriptional and epigenetic dysregulation. A single biopsy specimen containing a small amount of tumor tissue may not be representative of the whole tumor (72). In recent years, liquid biopsy techniques have been developed to collect samples from patients’ body fluids to obtain phenotypic, genetic, and transcriptomic information about the primary tumor (73). The primary forms of liquid biopsy include circulating tumor cells (CTCs), circulating tumor DNA (ctDNA), microRNA, and extracellular vesicles (74–77).

#### Circulating tumor cells

CTCs are malignant cells derived from either the primary or metastasis tumor that migrate into the systemic circulation, which represents a heterogeneous population of cells from the tumor. CTCs have been shown to be a reliable predictor of metastatic prostate cancer and breast cancer (78–81). A recent study conducted on HCC patients, of which 10 patients received anti-PD-1 therapy (9 with nivolumab and 1 with pembrolizumab), reported that all patients (*n* = 4) who did not have PD-L1+CTCs were non-responders (patients with PD or died within 6 months from initiating treatment). Meanwhile, all responders (patients with PR/SD) had PD-L1+CTCs detected at baseline. A longer OS was also found in PD-L1+CTC patients even after controlling for other factors [HR = 3.22 (95%CI 1.33-7.79), *p* = 0.01] (82). However, in a study of 47 HCC patients receiving a PD-1 inhibitor in combination with antiangiogenic therapy and radiotherapy, patients with low PD-L1+CTCs at baseline had a higher ORR (56.5% vs. 16.7%, *p* = 0.007) and longer OS (not reached vs. 10.8 months, *p* = 0.001) than those with high PD-L1+CTCs (83), indicating that CTC is still a controversial biomarker for predicting the treatment response to immunotherapy in HCC. As a result, larger sample studies are required to further explore its predictive value. Meanwhile, an extremely rare frequency of CTCs has been found in the circulation. All of these reasons make the detection of CTCs in the early stage of disease challenging (84).

#### Circulating tumor DNA

Circulating tumor DNA (ctDNA) can arise in the bloodstream of cancer patients as a result of tumor cell apoptosis or necrosis (85). ctDNA contains cancer-associated molecular characteristics, which allow its discrimination from total normal circulating cellular free DNA (86–88). In a subset of GO30140 arm A of 45 patients, higher ctDNA levels at baseline were associated with an increased baseline tumor burden (*p* < 0.03). After 3 cycles of treatment, ctDNA turned negative in 70% (CR), 27% (PR), 9% (SD), and 0% (PD) of patients, respectively. Patients with ctDNA cleared after 3 cycles of treatment showed longer PFS compared with those still present (6.5 vs. not reached months, *p* < 0.00029) (89). Patients with lower copy number variations (CNVs) in cell-free DNA risk score were also found to have longer OS and PFS in the ICI-treated cohort (90). Moreover, tumor mutational burden (TMB) evaluated by ctDNA was reported to be highly consistent with TMB detected by tissues (91), suggesting that ctDNA analysis could be an alternative option to evaluate TMB prior to immunotherapy in aHCC patients to whom tissue biopsy was not recommended if necessary. **Table 1** provides a brief overview of the biomarkers in peripheral blood for HCC immunotherapy.

**Table 1:** Predictive or prognostic biomarkers in peripheral blood for HCC immunotherapy.

Biomarkers	Treatment	Line of treatment	Number of detected patients	Cutoff value	Outcomes	Year	Ref.
NLR and PLR	Nivolumab (CheckMate 459: NCT02576509)	First-line or previously treated with sorafenib	NLR (*N* = 242); PLR (*N* = 243)	Tertile	Baseline lower NLR and PLR were associated with CR/PR and better OS	2020	(42)
	Nivolumab	Second-line (*N* = 129); third- or later-line (*N* = 65)	Baseline NLR (*N* = 194); dynamic NLR (*N* = 194)	NLR = 3	Patients with baseline NLR ≥3 had poorer PFS and OS; NLR increased rapidly in patients developing HPD; NLR increase at 4 weeks was associated with an increased risk of death, especially among patients with baseline NLR ≥3	2021	(43)
	Mono-immunotherapy and combination therapy	49% of patients with second-line therapy	Monotherapy (*N* = 310); combination therapy (*N* = 52)	NLR = 5; PLR = 300	Patients with higher NLR (≥5) and PLR (≥300) at baseline were reported having a higher incidence of PVT, higher ECOG performance status, more advanced BCLC stage, and shorter PFS and OS	2022	(44)
	Nivolumab	First-line (*N* = 66); subsequent-line (*N* = 37)	*N* = 103	NLR = 5; PLR tertiles	The combination of high NLR and PLR was found associated with an eightfold increased risk of death	2020	(40)
AFP and CRP	Nivolumab (*N* = 67); pembrolizumab (*N* = 32)	First-line (*N* = 13); subsequent-line (*N* = 86)	*N* = 99	AFP = 400 μg/L	Baseline AFP <400 μg/L was associated with better treatment response and longer PFS	2020	(52)
	Mono-immunotherapy and combined therapy	Training cohort: first-line (*N* = 82), subsequent-line (*N* = 108) Validation cohort: first-line (*N* = 35), subsequent-line (*N* = 67)	Training cohort (*N* = 190); validation cohort (*N* = 102)	AFP ≥100 ng/ml; CRP ≥1 mg/dl	Baseline serum AFP ≥100 ng/ml and CRP ≥1 mg/dl were independently associated with worse DCR and OS	2022	(53)
	TKI plus immunotherapy combination and lenvatinib monotherapy	Unknown	Combination cohort (*N* = 108); lenvatinib-treated cohort (*N* = 72)	AFP ≥100 ng/ml; CRP ≥1 mg/dl	Patients with baseline serum AFP ≥100 ng/ml and CRP ≥1 mg/dl showed worse OS and a trend toward lower ORR and DCR in the combination and the lenvatinib-treated cohorts	2022	(54)
TGF-β	Combination of TGF-β inhibition and immunotherapy	Unknown	Transcriptomic analyses (*N* = 193); pathway analyses (*N* = 70)	Unknown	A highly activated TGF-β signature was significantly associated with fibrosis and activated stromal signatures; TGF-β signature subtypes were significantly associated with immune cell infiltration and T-cell exhaustion	2020	(59)
	Pembrolizumab (NCT02658019)	Second-line	*N* = 24	TGF-β = 200 pg/ml	Patients with baseline TGF-β <200 pg/ml presented higher OS and PFS	2019	(60)
CD137	Sintilimab plus IBI305 (NCT04072679)	First-line	*N* = 33	CD137 = 31.8 pg/ml	CD137 concentration was significantly higher in patients with CB than in patients with non-CB	2022	(71)
CTC	Nivolumab (*N* = 9); pembrolizumab (*N* = 1)	First-line and subsequent-line	*N* = 10	Unknown	All patients (*n* = 4) who did not have PD-L1+CTCs were non-responders; meanwhile, all responders had PD-L1+CTCs detected at baseline; PD-L1+CTCs patients had longer OS after controlling for other factors	2020	(82)
	PD-1 inhibitor combined with radiotherapy and antiangiogenic therapy	First-line and subsequent-line	*N* = 47	2 PD-L1+CTCs	Patients with low PD-L1+CTCs at baseline had a higher ORR and longer OS than those with high PD-L1+CTCs	2022	(83)
ctDNA	Atezolizumab plus bevacizumab	First-line	*N* = 45	70.6 mean tumor molecules/ml of plasma (MTM/ml)	Higher ctDNA levels at baseline were associated with an increased baseline tumor burden; patients with ctDNA that cleared after 3 cycles of treatment showed longer PFS	2020	(89)
	Combination therapy of PD-1 inhibitor with lenvatinib and immune monotherapy	First-line and subsequent-line	Combination therapy (*N* = 43); immune monotherapy (*N* = 108)	CNV risk score: 15.68	Patients with lower CNVs had longer OS and PFS in the immunotherapy cohort	2021	(90)

HCC, hepatocellular carcinoma; NLR, neutrophil-to-lymphocyte ratio; PLR, platelet-to-lymphocyte ratio; CR, complete response; PR, partial response; OS, overall survival; ORR, objective response rate; HPD, hyperprogressive disease; PVT, portal vein thrombosis; ECOG, Eastern Cooperative Oncology Group; BCLC, Barcelona Clinic Liver Cancer; AFP, alpha-fetoprotein; CRP, C-reactive protein; DCR, disease control rate; TKI, multitargeted tyrosine kinase inhibitor; CBR, clinical benefit response; CTC, circulating tumor cell; ctDNA, circulating tumor DNA; PD-1, programmed cell death protein 1; CNV, copy number variation.

## Tumor tissue-related biomarkers

### Tumor immune microenvironment

The HCC tumor immune microenvironment (TiME) is acknowledged for its immunosuppressive character. The crosstalk between tumor cells and the immune microenvironment promotes tumor proliferation, invasion, and metastasis (92). Recent advances in basic and translational research have shown that the different manifestations of the tumor microenvironment are closely related to the efficacy of immunotherapy, revealing that the TiME profile may be a valuable potential biomarker of immunotherapy (14).

Analyses of patients treated with atezolizumab plus bevacizumab in the GO30140 arm A cohort showed that responders (CR/PR) had a higher density of infiltrating CD8^+^ T cells, CD3^+^ T cells, and GZMB^+^CD3^+^ T cells in tumor areas than non-responders (SD/PD) (*p* = 0.007, *p* = 0.039, and *p* = 0.044, respectively). The high presence of several immune subsets, including CD8 and CD4 T cells, Tregs, B cells, and dendritic cells, also seemed to be associated with better response and longer PFS (93). In the IMbrave150 cohort, patients with a high density of intratumoral CD8^+^ T cells showed longer OS [HR = 0.29 (95% CI 0.14–0.61), *p* = 0.001] and PFS [HR = 0.54 (95% CI 0.29–1.00), *p* = 0.053] in atezolizumab plus bevacizumab compared with sorafenib. The reason may be that blockade of the PD-1/PD-L1 axis could restore the antitumor immunity induced by CD8^+^ lymphocytes in tumors (94). The results above demonstrated that patients with pre-existing immunity seemed to possess improved clinical outcomes to combination therapy. An exploratory research in CheckMate 040 analyzed the levels of multiple inflammation biomarkers and their association between treatment response and survival to nivolumab in patients previously treated with or without sorafenib (42). The results showed that patients with CR/PR had higher CD3^+^ T cells compared with those with SD (*p* = 0.03). Those with higher tumor-infiltrating CD3^+^ and CD8^+^ T cells showed a trend toward improved OS (both *p* = 0.08).

Macrophages are major components of the TiME, which can be classified into two main subtypes: the classically activated macrophages (M1 macrophages) with pro-inflammatory functions and the alternatively activated macrophages (M2 macrophages) with immunosuppressive functions (95). Several tumor-promoting roles, such as immune suppression, cancer invasion and metastasis, angiogenesis, maintenance of cancer cell stemness, and drug resistance, have been attributed to these tumor-associated macrophages (TAMs), especially M2 macrophages (96, 97). A previous study had shown that high levels of M2 macrophages have been associated with poor prognosis in patients with HCC (98). Although the expression of CD68^+^ and CD163^+^ (M2 macrophages) cells has no association with either treatment response or OS in the CheckMate 040 subgroup (42), the other study reported that a higher density of M1 macrophages (CD68^+^CD163^−^) in the stroma was associated with better efficacy and longer PFS (M1 macrophages low vs. high: 11.4 vs. 3.0 months, *p* = 0.024) and OS (M1 macrophages low vs. high: undefined vs. 17.5 months, *p* = 0.046) (71). Therefore, a better understanding of the mechanisms underlying the function of TAMs is necessary for the development of novel TAM-targeting immunological interventions, which may provide promising therapeutic approaches for HCC patients. Collectively, immunocytes with different functions can directly reflect tumor immune status. This advantage makes it become the main research direction at present. More mechanistic research is expected to be further carried out.

### Signaling pathway and gene signature

As important regulatory factors in tumor progression and the immune environment, the correlation between molecular features and treatment efficacy has become the focus of recent research. In the GO30140 group A cohort, pathways and immune subsets were identified by genome-wide differentially expressed genes (DEGs), gene set enrichment analysis (GSEA), and xCell analyses. An atezolizumab + bevacizumab response signature (ABRS) consisting of the top 10 genes from the DEG analyses (namely, *CXCR2P1*, *ICOS*, *TIMD4*, *CTLA4*, *PAX5*, *KLRC3*, *FCRL3*, *AIM2*, *GBP5*, and *CCL4*) was found consistently higher in patients with CR/PR than in those with SD/PD, as well as the T effector (Teff) signature (*CXCL9*, *PRF1*, and *GZMB*). Patients with a high expression of these markers had longer PFS than those with low expression [ABRS: HR = 0.51 (95% CI 0.30–0.87), *p* = 0.013; Teff signature: HR = 0.46 (95% CI 0.27–0.78), *p* = 0.0035], which was further validated in the IMbrave150 cohort. Patients with high ABRS or the Teff signature showed improved PFS [ABRS: HR = 0.49 (95% CI 0.25–0.97), *p* = 0.041; Teff signature: HR = 0.52 (95% CI 0.28–0.99), *p* = 0.047] and OS [ABRS: HR = 0.26 (95% CI 0.11–0.58), *p* = 0.0012; Teff signature: HR = 0.24 (95% CI 0.11–0.5), *p* = 0.0002] when treated with atezolizumab + bevacizumab compared with sorafenib. Signature analysis in IMbrave150 revealed that a low ratio of Treg/Teff signatures was associated with improved PFS [HR = 0.42 (95% CI 0.22–0.79), *p* = 0.007] and OS [HR = 0.24 (95% CI 0.11–0.54), *p* = 0.0006] when treated with atezolizumab + bevacizumab compared with sorafenib. As for the mutation landscape, *TERT* promoter mutations were observed in 56.2% of patients in the IMbrave150 trial. The benefit of atezolizumab + bevacizumab was more pronounced in patients with *TERT*-mutant than in the sorafenib group [PFS: HR = 0.61 (95% CI 0.33–1.10), *p* = 0.047; OS: HR = 0.38 (95% CI 0.16–0.89), *p* = 7.8 × 10^−5^] (93).

Hyperactive Wnt/β‐catenin signaling is implicated in the initiation and progression of various types of cancer, which may be related to the exclusion of CD8^+^ cells in tumor tissues in melanoma cases (99). *CTNNB1* which is involved in the Wnt/β-catenin signaling pathway is a prevalent mutation gene in HCC (100). Approximately 11%–41% of liver malignancies harbor *CTNNB1*-activating mutations (93, 101–104). Several studies have shown that β‐catenin signaling may mediate the immune escape of cancer cells and the resistance to ICIs (99, 105, 106). In the IMbrave150 trial, patients with wild-type *CTNNB1* showed greater treatment effect with atezolizumab + bevacizumab than sorafenib [PFS: HR = 0.45 (95% CI 0.27–0.86), *p* = 0.0086; OS: HR = 0.42 (95% CI 0.19–0.91), *p* = 3 × 10^−4^) (93). A small sample cohort of 34 patients who were treated with anti-PD-1 monotherapy with or without previous treatment with sorafenib found that although patients with negative Wnt/β-catenin activation, high CD8^+^ TIL infiltration, and high PD-L1-CPS showed higher DCR, PFS, and OS in the univariate analysis, no significant difference was presented after the multivariate analysis. However, the combination of these factors well stratified the survival in both PFS (*p* < 0.0001) and OS (*p* = 0.0048), suggesting that in patients lacking β-catenin activation, blockade of the PD-1/PD-L1 axis might overcome the presence of exhausted TILs (107). Harding et al. (108) studied 27 HCC patients treated with ICIs (both monotherapy and combination therapy). The results showed that all patients with Wnt pathway alterations had PD at the first interval scan, whereas 9 of 17 non-Wnt pathway-altered patients had durable disease (SD ≥4 months) or better as the best response (*p* < 0.009). Wnt-activated patients presented shorter mPFS (2.0 vs. 7.4 months, *p* < 0.0001) and numerically shorter OS (9.1 vs. 15.2 months, *p* = 0.11). Lin et al. analyzed the same cohort and verified the results (109). The role of Wnt/β-catenin signaling as a biomarker has been verified in a number of studies, which can be further explored as a powerful factor in a prospective prediction model.

### PD-L1 expression and tumor mutational burden

PD-L1 has been reported as an indicator of anti-PD-1/PD-L1 treatment in several cancer types (110–112). However, the predictive role of PD-L1 expression in HCC immunotherapy remains controversial. In the KEYNOTE-224 trial, PD-L1 expression calculated by the combined positive score (CPS, cutoff = 1) was found to be associated with improved ORR and PFS in responders (CR/PR), whereas PD-L1 expression calculated by the tumor proportion score (TPS, cutoff = 1%) has no predictive value as CPS (8). In the CheckMate 459 trial, although patients with baseline PD-L1 expression ≥1% had higher ORR in the nivolumab group (28% vs. 12%), no difference was observed in PFS and OS (113). In the dose-escalation and dose-expansion cohort of Checkmate 040, TPS did not have an apparent predictive effect on the response rate (9). The same result was found in the nivolumab and ipilimumab combined cohort of CheckMate 040 (114). When PD-L1 was detected by the expression of CD274 (PD-L1 messenger RNA) in the G030140 arm A group and in the IMbrave150 trial, it was found to be higher in patients with CR/PR than in patients with SD/PD. Patients with high levels of CD274 also showed longer PFS than those with low expression [HR = 0.42 (95% CI 0.25–0.72), *p* = 0.0011] (93). Patients with high expression of CD274 showed improved PFS and OS in the combination therapy group than in the sorafenib group. In conclusion, the predictive value of PD-L1 is limited in HCC immunotherapy.

Previous studies in melanoma and NSCLC showed that higher TMB was associated with higher tumor responsiveness to PD-1/PD-L1 immunotherapy (115–117). Nonetheless, the value of TMB as an objective biomarker in immunotherapy in HCC remains indefinite. Xie et al. analyzed the HCC cohort with immunotherapy from The Cancer Genome Atlas and found that higher TMB was associated with the immune microenvironment diversification and worse prognosis (118). In the GO30140 biomarker exploration study, ORR was found to be higher in patients with high TMB than in those with median or low levels in 76 patients in arm A (56% vs. 17% vs. 35%), while PFS has no difference in all the groups (high vs. median vs. low = 13.6 vs. 5.9 vs. 7.9 months). However, no association between TMB and treatment response or survival was found in the combination therapy group in the IMbrave150 cohort (93). The same result was found in another anti-PD-1 treatment cohort (52). The potential reason may be attributed to the generally low level of TMB in HCC (median TMB in HCC was only 4.08) (108). **Table 2** provides a brief overview of the biomarkers in tumor tissues for HCC immunotherapy.

**Table 2:** Predictive or prognostic biomarkers in tumor tissues for HCC immunotherapy.

Biomarker	Treatment	Line of treatment	Number of detected patients	Outcome	Year	Ref.
SCD8^+^ T cells, CD3^+^ T cells, and GZMB^+^CD3^+^ T cells	Atezolizumab plus bevacizumab (GO30140: NCT02715531)	First-line	*N* = 61	Responders (CR/PR) had a higher density of infiltrating CD8**^+^** T cells, CD3**^+^** T cells, and GZMB**^+^** CD3**^+^** T cells in tumor areas than non-responders (SD/PD)	2022	(93)
Immune subsets	Atezolizumab plus bevacizumab (GO30140: NCT02715531)	First-line	*N* = 90	High presence of several immune subsets, including CD8**^+^** and CD4**^+^** T cells, Tregs, B cells, and dendritic cells, associated with better response and longer PFS		
Intratumoral CD8^+^ T cells	Atezolizumab plus bevacizumab vs. sorafenib (IMbrave150: NCT03434379)	First-line	Atezolizumab plus bevacizumab (*N* = 119); sorafenib (*N* = 58)	Patients with a high density of intratumoral CD8**^+^** T cells showed longer OS and PFS with atezolizumab plus bevacizumab compared with sorafenib		
CD3^+^ T cells and CD8^+^ T cells	Nivolumab (CheckMate 459: NCT02576509)	First-line or previously treated with sorafenib	CD3**^+^** T cells (*N* = 189), CD8**^+^** T cells (*N* = 192)	Higher CD3**^+^** T cells were associated with patients with CR/PR compared with patients with SD Patients with higher tumor-infiltrating CD3^+^ T cells and CD8^+^ T cells showed an improved OS trend	2022	(42)
CD68^+^ and CD163^+^ (M2 macrophages)	Nivolumab (CheckMate 459: NCT02576509)	First-line or previously treated with sorafenib	*N* = 135	CD68**^+^** and CD163**^+^** cells have no association with either response or OS	2021	
CD68^+^ and CD163^−^ (M1 macrophages)	Sintilimab plus IBI305 (NCT04072679)	First-line	*N* = 33	Higher density of M1 macrophages (CD68**^+^** CD163**^+^**) in the stroma is associated with better efficacy and longer PFS and OS	2022	(71)
ARBS and Teff	Atezolizumab plus bevacizumab and atezolizumab plus bevacizumab vs. sorafenib (GO30140: NCT02715531; IMbrave150: NCT03434379)	First-line	GO30140 (*N* = 90); IMbrave150 (atezolizumab plus bevacizumab, *N* = 119; sorafenib, *N* = 58)	Higher expression of ABRS and Teff had better treatment response and longer PFS High expression of ABRS or the Teff signature showed improved PFS and OS when treated with atezolizumab + bevacizumab vs. sorafenib	2022	(93)
Treg/Teff	Atezolizumab plus bevacizumab and atezolizumab plus bevacizumab vs. sorafenib (GO30140: NCT02715531; IMbrave150: NCT03434379)	First-line	GO30140 (*N* = 90); IMbrave150 (atezolizumab plus bevacizumab, *N* = 119; sorafenib, *N* = 58)	Low ratio of Treg/Teff signatures was associated with improved PFS and OS with atezolizumab + bevacizumab vs. sorafenib		
*TERT* promoter mutation	Atezolizumab plus bevacizumab vs. sorafenib (IMbrave150: NCT03434379)	First-line	IMbrave150 (atezolizumab plus bevacizumab, *N* = 85; sorafenib, *N* = 45)	Patients with *TERT*-mutant tumors showed longer PFS and OS in the atezolizumab + bevacizumab group than in the sorafenib group		
Wnt/β-catenin	Atezolizumab plus bevacizumab vs. sorafenib (IMbrave150: NCT03434379)	First-line	IMbrave150 (atezolizumab plus bevacizumab, *N* = 85; sorafenib, *N* = 45)	Patients with wild-type *CTNNB1* showed greater treatment effects from atezolizumab + bevacizumab vs. sorafenib than those with *CTNNB1* mutations		
	Anti-PD-1 monotherapy	First-line or previously treated with sorafenib	*N* = 34	The combination of Wnt/β-catenin activation, high CD8^+^ TIL infiltration and high PD-L1-CPS well stratified the survival of the patients in both PFS and OS	2021	(107)
	Mono-immunotherapy and combination therapy (NCT01775072)	First-line or previously treated with sorafenib	*N* = 27	Patients with Wnt pathway alterations had worse treatment response, shorter mPFS, and numerically shorter OS	2019	(108)
PD-L1	Pembrolizumab (KEYNOTE-224: NCT02702414)	Second-line	*N* = 52	CPS was associated with improved ORR and PFS in responders (CR/PR), whereas TPS has no predictive value	2018	(8)
	Nivolumab or nivolumab plus ipilimumab (CheckMate 040: NCT01658878)	First- or second-line (dose-escalation and dose-expansion phase)	Dose-escalation phase (*N* = 44); dose-expansion phase (*N* = 174)	Baseline tumor cell PD-1 status has no apparent effect on the response rate	2017	(9)
		Second-line (nivolumab plus ipilimumab cohort)	*N* = 148	Responses occurred regardless of PD-L1 expression	2022	(114)
	Nivolumab (CheckMate 459: NCT02576509)	First-line	Nivolumab (*N* = 366); sorafenib (*N* = 364)	Patients with baseline PD-L1 expression ≥1% had higher ORR in the nivolumab group	2021	(113)
	Atezolizumab plus bevacizumab and atezolizumab plus bevacizumab vs. sorafenib (GO30140: NCT02715531; IMbrave150: NCT03434379)	First-line	GO30140 (*N* = 90); Imbrave150 (atezolizumab plus bevacizumab, *N* = 119; sorafenib, *N* = 58)	PD-L1 detected by CD274 (PD-L1 messenger RNA) was higher in patients with CR/PR than SD/PD; patients with high expression of CD274 showed longer PFS in the combination therapy group. High expression of CD274 showed improved PFS and OS when treated with atezolizumab + bevacizumab vs. sorafenib	2022	(93)
TMB	Immunotherapy	Unknown	*N* = 377	Higher TMB was associated with the immune microenvironment diversification and a worse prognosis	2020	(118)
	Atezolizumab plus bevacizumab and atezolizumab plus bevacizumab vs. sorafenib (GO30140: NCT02715531; IMbrave150: NCT03434379)	First-line	GO30140 (*N* = 76); IMbrave150 (atezolizumab plus bevacizumab, *N* = 119; sorafenib, *N* = 58)	ORR was found higher in patients with high TMB than in those with median or low level in the GO30140 arm A; no association was found in the other analysis	2022	(93)
	Nivolumab or pembrolizumab	First-line or subsequent-line	*N* = 15	TMB could not predict treatment response and PFS	2020	(52)

HCC, hepatocellular carcinoma; ARBS, atezolizumab + bevacizumab response signature (including *CXCR2P1*, *ICOS*, *TIMD4*, *CTLA4*, *PAX5*, *KLRC3*, *FCRL3*, *AIM2*, *GBP5*, and *CCL4*); Teff, T effector (including *CXCL9*, *PRF1*, and *GZMB*); CR, complete response; PR, partial response; SD, stable disease; PD, progressive disease; OS, overall survival; PFS, progression-free survival; PD-L1, programmed death ligand 1; CPS, combined positive score; TPS, tumor proportion score; ORR, objective response rate; PD-1, programmed death 1; TMB, tumor mutational burden.

## Gut microbiota

The gut microbiota is known to influence immune responses and even promote carcinogenesis, which supports its potential role as a biomarker in immunotherapy. Accumulated evidence has shown that the gut microbiota may predict immunotherapy efficacy in various cancer types (119–123). Several mechanisms have been reported such as modulating DNA damage, influencing oncogenesis or tumor suppression by metabolic processes (124), and inducing regulatory T-cell expansion and CD8^+^ T-cell attenuation (125). The above mechanisms finally inhibit antitumor immunity through mediating immune cells and cytokine production (126–130). Several studies have been conducted to explore the role of the gut microbiome in HCC immunotherapy in recent years. Zheng et al. (131) found that fecal samples from HCC patients treated with camrelizumab showed higher taxa richness and more gene counts of gut microbiome species, such as *Akkermansia* and *Ruminococcaceae*, than from non-responders. Meanwhile, the dissimilarity of beta diversity became prominent as early as 6 weeks, which indicated that the gut microbiome might be used for early prediction for anti-PD-1 immunotherapy after treatment initiation. However, only 8 patients were enrolled in this study. Zhao et al. (132) conducted an analysis of 65 patients with advanced hepatobiliary cancer receiving anti-PD-1 treatment to explore the potential mechanism. The results showed that the clinical benefit response (CBR) group (patients with CR, PR, or SD ≥6 months) had more taxa enrichment than the non-clinical benefit (NCB) group (patients with SD <6 months or PD). *Lachnospiraceae bacterium-GAM79* and *Alistipes spMarseille-P5997* were significantly enriched in the CBR group (74 vs. 40 taxa). Patients with a higher abundance of *Ruminococcus calidus* and *Erysipelotichaceae bacterium-GAM147* presented longer PFS and OS. During treatment, the gut microbiome composition in the CBR group remained stable, while in the NCB group, the microbial diversity seemed to decrease. Fecal microbiota transplantation (FMT) from donors who achieved CR/PR for a long duration treated with anti-PD-1 therapy to patients who were refractory to immunotherapy was reported to increase intratumor lymphocyte infiltration in patients with poor efficacy in melanoma (133, 134). A study conducted in a mouse model showed that FMT could significantly enhance the therapeutic efficacy of ICIs in syngeneic tumor models by increasing tumor-infiltrating IFN-γ^+^CD8^+^ T cells and the tumor suppression effect (135). All these findings demonstrated that the gut microbiome might be an effective biomarker to predict the clinical response and survival benefit of immunotherapy in HCC. Its predictive prospect is worth anticipating.

## Immune-related adverse events

Immunotherapy will inevitably result in irAEs, which are defined as side effects with potential immunological basis and require more frequent monitoring and possible treatment with systemic steroids (136). Mono-immunotherapy conducted by nivolumab in CheckMate 040 and CheckMate 459 resulted in 22%–25% grade 3–5 AEs (9, 137). For pembrolizumab in KEYNOTE-240 and KEYNOTE-224, grade 3–5 AEs were about 52% and 26% (8, 138). As for the combination of immune and targeted therapy, grade 3–5 AEs were about 50%–60% in IMbrave150, GO30140, and ORIENT-32 (12, 139, 140). Several studies conducted in solid malignancies have demonstrated a positive association between irAEs with improved clinical outcomes, such as melanoma, urothelial cancer, renal cell carcinoma, NSCLC, and gastric cancer (141–146). A consistent result was found in a retrospective cohort study of 168 patients with aHCC. In this study, patients with grade ≥3 irAEs demonstrated improved ORR and DCR than those with no irAEs (ORR: 50% vs. 11.3%, *p* = 0.002; DCR: 87.5% vs. 28.2%, *p* < 0.001). The median PFS and OS in patients with grade ≥3 irAEs and grade 1–2 irAEs were significantly longer than in patients with no irAEs (PFS: 8.5 vs. 3.6 vs. 1.3 months, *p* < 0.001; OS: 26.9 vs. 14.0 vs. 4.6 months, *p* < 0.001). Patients with more severe and multisystem (two or more systems) irAEs have a better prognosis (147). The mechanisms are still unclear. Possibly, patients who experience more serious irAEs could have higher T-cell activity and experience better antitumor outcomes. Other possible mechanisms may rely on the potential similar pathway shared by adverse events and the ICIs. Thus, the occurrence of adverse events may reflect that the relative pathway has been inhibited at a high level, which certainly led to better efficacy (148).

## The etiology of hepatocellular carcinoma

Over 90% of HCC cases occur in the setting of chronic liver disease. The major risk factors for HCC are chronic infection with hepatitis B virus (HBV) or hepatitis C virus (HCV), heavy alcohol intake, excess body weight, diabetes, or non-alcoholic fatty liver disease (NAFLD) (1).

The response of HCC induced by various etiologies to immunotherapy may differ. Chun et al. reported that Treg and CD8^+^ resident memory T cells (TRM) were enriched in HBV-related HCC. Treg and TRM from HBV-related HCC expressed more PD-1 and were functionally more suppressive and exhausted than those from non-viral-related HCC, which could be reversed by anti-PD-1 blockade (149). A meta-analysis included eight systemic therapies cohorts to evaluate the impact of targeted and immune therapies according to different HCC etiologies. Among them, five were TKI/anti-VEGF cohorts (REACH, REACH-2, METIV-HCC, CELESTIAL, and JET-HCC) and three were immunotherapy cohorts (Checkmate 459, IMbrave150, and KEYNOTE-240). Patients with viral-related HCC presented significantly better OS than those with non-viral-related HCC (*p* = 0.0259) in immunotherapy. Efficacy was similar in HBV- and HCV-related HCC [HR = 0.64 (95% CI 0.49–0.83) vs. HR = 0.68 (95% CI 0.47–0.98)]. No impact of etiology was observed in TKI/anti-VEGF therapies (150). In another meta-analysis, the presence of viral infection had a significant interaction with the ICI efficacy in HBV-infected HCC (*p*_interaction_ = 0.016) but not in HCV-infected HCC (*p*_interaction_ = 0.081) (151). The potential reason may be that patients with HCV–HCC were rich in Tregs and M2 macrophages and had an upregulated expression of CTLA4 and other immunosuppressive molecules (152–154), and the expression of negative co-stimulatory signals may contribute to treatment resistance. Meanwhile, unlike HBV-related HCC, the function of HCV-specific CD8^+^ T cells did not recover after PD-1/PD-L1 blockade (155). However, contrary to the above results, Ho et al. found that the ORR between viral-infected and uninfected patients showed no clinical difference when treated with PD-1/PD-L1 inhibitors, which means that viral status is not suitable to be used as a criterion to select patients for immunotherapy (156). Considering that the meta-analyses were not based on individual patient’s data and the trials included were heterogeneous in terms of treatment line and control arm, whether patients with viral infection respond better to immunotherapy than those without infection requires further research.

NAFLD has become an emerging risk factor for HCC over the past decade (157). In a retrospective study with 79 patients [15 patients in the non-alcoholic steatohepatitis (NASH) cirrhosis-related HCC group and 64 patients in the HCC group without NASH cirrhosis], there were significantly higher rates of PD as the best response to immunotherapy in patients with HCC and NASH cirrhosis compared with those without NASH cirrhosis (46.7% vs. 10.9%, *p* = 0.004) (158). A relevant mechanism research found that the exhausted, unconventionally activated CD8^+^PD1^+^ T cells progressively accumulated in NASH-affected livers. However, in mice with NASH but without HCC, preventive CD8^+^ T-cell depletion significantly decreased the incidence of HCC. Meanwhile, preventive anti-PD-1 treatment in NASH mice increased CD8^+^PD1^+^ T cells and also caused a marked increase in cancer incidence, which means CD8^+^PD1^+^ T cells from patients with NAFLD or NASH might help induce NASH–HCC, rather than invigorating or executing immune surveillance (159). Collectively, NASH–HCC might be less responsive to immunotherapy, probably owing to NASH-related aberrant T-cell activation causing tissue damage that leads to impaired immune surveillance. **Table 3** provides a brief overview of the biomarkers of other types for HCC immunotherapy.

**Table 3:** Predictive or prognostic biomarkers of other types for HCC immunotherapy.

Biomarker	Treatment	Line of treatment	Number of detected patients	Outcome	Year	Ref.
Gut microbiota	Anti-PD-1-based systemic therapy (NCT03892577) (NCT03895970) (NCT04010071)	Not mentioned	HCC (*N* = 30)	Baseline gut microbiome diversity is associated with a favorable response to anti-PD-1 treatment; higher diversity and relative abundance of taxa might be a protective factor against irAEs	2021	(132)
	SHR-1210 (NCT02989922)	Second-line	*N* = 8	Responders showed higher taxa richness and more gene counts of gut microbiome species than non-responders	2019	(131)
irAEs	Monotherapy and combination therapy	First-line or subsequent-line	*N* = 168	Patients with more severe irAEs and multisystem (two or more systems) involvement have a better prognosis	2021	(147)
Etiology	CheckMate 459 (NCT02576509) IMbrave150 (NCT03434379) KEYNOTE-240 (NCT02702401)	First-line or second-line	*N* = 1,656 Meta-analysis	Immunotherapy is less effective in non-viral etiologies than in viral-related HCC. The effect of ICIs was remarkably similar in HBV- and HCV-related HCC	2021	(150)
	CheckMate 459 (NCT02576509) IMbrave150 (NCT03434379) KEYNOTE-240 (NCT02702401)	First-line or second-line	*N* = 1,656 Meta-analysis	The presence of viral infection had a significant interaction with the ICI efficacy in HBV-infected but not in HCV-infected patients	2021	(151)
	Monotherapy and combination therapy	First-line or second-line	*N* = 567 Meta-analysis	ORR between virally infected and uninfected patients showed no clinically meaningful difference	2020	(156)
	Atezolizumab, nivolumab, pembrolizumab	Unknown	*N* = 79	NASH-related HCC patients showed significantly higher rates of disease progression as the best response to immunotherapy compared with those without NASH cirrhosis	2022	(158)
	Monotherapy and combination therapy	First-line or subsequent-line	*N* = 248 (118 patients in the validation cohort)	NAFLD is associated with a worse outcome in patients with HCC treated with PD(L)1-targeted immunotherapy	2021	(159)

HCC, hepatocellular carcinoma; PD-1, programmed death 1; irAEs, immune-related adverse events; HBV, hepatitis B virus; HCV, hepatitis C virus; NASH, non-alcoholic steatohepatitis; NAFLD, non-alcoholic fatty liver disease.

## Conclusion

We have concluded an exploratory research on biomarkers as immunotherapy for HCC. As biomarkers detected from peripheral blood, NLR, PLR, and CRAFITY (CRP and AFP in Immunotherapy) score, which are not only easy to be collected but also with high prognostic value supported by a large sample research, are of great significance in the future construction of predictive models. The gene signature and the tumor immune microenvironment have the ability to precisely reflect the pre-existing immunity in baseline tumor tissues, which have shown potential predictive value to drive the clinical activity of immunotherapy in aHCC in the IMbrave150 trial. The gut microbiota and irAEs which were found to be potential biomarkers in immunotherapy are now being further analyzed and are expected to be explored in the future. Fecal microbiota transplantation has been even developed into a combination treatment method and has shown great promise to increase immunotherapy efficacy. CD137 and other cytokines are potential predictive factors that need to be verified in large sample cohorts. Integrative multiparametric approaches that combine peripheral markers, the tumor microenvironment, and immune signatures appear to be the most comprehensive way to assess treatment outcomes and seem to be promising in the future.

## Author contributions

XP: conceptualization, data curation, figure preparation, and writing—original draft preparation. CG: writing—review and editing. WZ: writing—review and editing, project administration, and funding acquisition. AZ: conceptualization, writing—original draft preparation, writing—review and editing, project administration, and funding acquisition. All authors contributed to the article and approved the submitted version.

## Glossary

**Table:**

HCC	hepatocellular carcinoma
aHCC	advanced hepatocellular carcinoma
TKIs	multitargeted tyrosine kinase inhibitors
OS	overall survival
ICIs	immune checkpoint inhibitors
FDA	Food and Drug Administration
PD-1	programmed death 1
NMPA	National Medical Products Administration
ORR	objective response rate
irAEs	immune-related adverse effects
NLR	neutrophilto-lymphocyte ratio
PLR	platelet-to-lymphocyte ratio
TACE	transarterial chemoembolization
CR	complete response
PR	partial response
PD	progressive disease
PFS	progression-free survival
HR	hazard ratio
HPD	hyperprogressive disease
PVT	portal vein thrombosis
ECOG	Eastern Cooperative Oncology Group
BCLC	Barcelona Clinic Liver Cancer
VEGF	vascular endothelial growth factor
AFP	alpha-fetoprotein
CRP	C-reactive protein
SD	stable disease
DCR	disease control rate
TGF-β	transforming growth factor-β
TNFRSF9	TNF receptor superfamily member 9
PD-L1	programmed death ligand 1
DFS	disease-free survival
CB	clinical benefit
CTC	circulating tumor cell
ctDNA	circulating tumor DNA
CNV	copy number variation
TiME	tumor immune microenvironment
TAM	tumor-associated macrophage
DEG	differentially expressed gene
GSEA	gene set enrichment analysis
ABRS	atezolizumab + bevacizumab response signature
Teff	T effector
TMB	tumor mutational burden
CPS	combined positive score
TPS	tumor proportion score
CBR	clinical benefit response
NCB	non-clinical benefit
FMT	fecal microbiota transplantation
HBV	hepatitis B virus
HCV	hepatitis C virus
NAFLD	non-alcoholic fatty liver disease
NASH	nonalcoholic steatohepatitis
